# Antioxidant-Based Therapies in Male Infertility: Do We Have Sufficient Evidence Supporting Their Effectiveness?

**DOI:** 10.3390/antiox10020220

**Published:** 2021-02-02

**Authors:** Angela Maria Amorini, Ilaria Listorti, Gabriele Bilotta, Romina Pallisco, Miriam Wissam Saab, Renata Mangione, Benedetta Manca, Giacomo Lazzarino, Barbara Tavazzi, Giuseppe Lazzarino, Pasquale Bilotta

**Affiliations:** 1 Department of Biomedical and Biotechnological Sciences, Division of Medical Biochemistry, University of Catania, Via S. Sofia 97, 95123 Catania, Italy; amorini@unict.it (A.M.A.); mirisaab@gmail.com (M.W.S.); 2 Alma Res Fertility Center, Laboratory of Biology and Embriology, Via Parenzo 12, 00199 Rome, Italy; laboratorio@almares.it (I.L.); bilotta.oblomov@gmail.com (G.B.); romina.pallisco@almares.it (R.P.); 3 Department of Basic Biotechnological Sciences, Intensive and Perioperative Clinics, Catholic University of the Sacred Heart of Rome, Largo F. Vito 1, 00168 Rome, Italy; renata.mangione@unicatt.it; 4 Department of Pharmacy and Biotechnology (FaBiT), University of Bologna, Via Irnerio 48, 40126 Bologna, Italy; benedetta.manca4@unibo.it; 5 UniCamillus—Saint Camillus International University of Health Sciences, Via di Sant’Alessandro 8, 00131 Rome, Italy; 6 Fondazione Policlinico Universitario A. Gemelli IRCCS, Largo A. Gemelli 8, 00168 Rome, Italy; 7 LTA-Biotech srl, Viale Don Orione, 3D, 95047 Paternò, Italy; 8 Alma Res Fertility Center, Laboratory of Obstetrics and Ginecology, Via Parenzo 12, 00199 Rome, Italy; pasquale.bilotta@almares.it

**Keywords:** male infertility, oxidative/nitrosative stress, antioxidants, seminal plasma, spermatozoa

## Abstract

Under physiological conditions, reactive oxygen species (ROS) play pivotal roles in various processes of human spermatozoa. Indeed, semen requires the intervention of ROS to accomplish different stages of its maturation. However, ROS overproduction is a well-documented phenomenon occurring in the semen of infertile males, potentially causing permanent oxidative damages to a vast number of biological molecules (proteins, nucleic acids, polyunsaturated fatty acids of biological membrane lipids), negatively affecting the functionality and vitality of spermatozoa. ROS overproduction may concomitantly occur to the excess generation of reactive nitrogen species (RNS), leading to oxidative/nitrosative stress and frequently encountered in various human pathologies. Under different conditions of male infertility, very frequently accompanied by morpho-functional anomalies in the sperm analysis, several studies have provided evidence for clear biochemical signs of damages to biomolecules caused by oxidative/nitrosative stress. In the last decades, various studies aimed to verify whether antioxidant-based therapies may be beneficial to treat male infertility have been carried out. This review analyzed the results of the studies published during the last ten years on the administration of low-molecular-weight antioxidants to treat male infertility in order to establish whether there is a sufficient number of data to justify antioxidant administration to infertile males. An analysis of the literature showed that only 30 clinical studies tested the effects of the administration of low-molecular-weight antioxidants (administered as a single antioxidant or as a combination of different antioxidants with the addition of vitamins and/or micronutrients) to infertile males. Of these studies, only 33.3% included pregnancy and/or live birth rates as an outcome measure to determine the effects of the therapy. Of these studies, only 4 were case–control studies, and only 2 of them found improvement of the pregnancy rate in the group of antioxidant-treated patients. Additionally, of the 30 studies considered in this review, only 43.3% were case–control studies, 66.7% enrolled a number of patients higher than 40, and 40% carried out the administration of a single antioxidant. Therefore, it appears that further studies are needed to clearly define the usefulness of antioxidant-based therapies to treat male infertility.

## 1. Introduction

Infertility is a far-reaching condition affecting 8–12% of reproductive-age couples worldwide [1]. It is defined as the inability to have conception after 12 months of unprotected sexual intercourse [2]. Historically, females have often been indicated as the sole ones responsible for pregnancy failures. During the last decades, thanks to the increasing number of epidemiological studies regarding infertility, it came forth into the light that the male factor is implicated in around 50% of cases of conceiving problems. Nowadays, among couples affected by this condition, the male factor is considered the unique cause in ≈20% of all cases, together with a female factor co-contribution in the remaining 30% of cases [3,4,5]. The etiological causes of male infertility comprise a wide variety of factors, ranging from infections, anatomical and genetic abnormalities, neurological disease, environmental and lifestyle [6,7,8] and psychosocial factors, defining this condition as a multifactorial disease. In this context, it is worth recalling that even the COVID-19 pandemic has recently been included as a new external factor potentially negatively affecting couple fertility [9,10]. Indeed, since SARS-Cov-2 viral infection is responsible for systemic inflammatory processes, also leading to increased oxidative/nitrosative stress [11], it has been hypothesized that it may alter various fertility-related biochemical processes, thus resulting in decreased fertilization capacities of both males and females [12].

### 1.1. Clinical Classification/Diagnosis of Male Infertility

Male infertility is generally characterized by morpho-functional anomalies in the sperm analysis, including low sperm count, alteration of the sperm quality parameters (concentration, motility, morphology), or both. The main causes of male infertility are divided into: (1) extra-testicular (obstructive); (2) testicular (primary); (3) pretesticular (secondary); (4) idiopathic [13,14]. This last group, in whom the primary cause is not clearly manifest, accounts for 30–40% of infertile male patients [14].

Extra-testicular causes affect about 10–20% of infertile males and are mainly due to an impairment of sperm delivery or ejaculatory dysfunctions [15]. Sperm is transported, in anticipation of ejaculation, through three different parts of the male reproductive system, namely vasa deferens, epididymis and ejaculatory ducts. The absence of vasa deferens is a condition affecting 1–2% of all infertile males [16], caused by mutations on the cystic fibrosis transmembrane regulator gene CFTR or by abnormalities during embryogenesis [17]. The most common causes of obstruction of the vasa deferens are vasectomy and accidental injury during inguinal hernia surgical procedures. Epididymal obstructions are most commonly caused by infections (in which the etiological agents are gonococcus and chlamydia), epididymal cysts, trauma and the genetic condition known as Young’s syndrome [18,19]. The main causes of the obstruction of ejaculatory ducts can be both congenital, due to the compression of Müllerian or Wolffian ducts [20] and acquired, due to the insurgence of prostatic or seminal vesicle calculi or scar tissue formation [21]. The other main family of causes of extra-testicular infertility is represented by the ejaculatory dysfunctions, a heterogeneous group of disorders primarily linked with the conditions of premature ejaculation, anejaculation and retrograde ejaculation induced by pharmacological, anatomical or idiopathic factors [22].

Testicular infertility, also called primary hypogonadism, representing a set of congenital or acquired pathological conditions, affects about 30–40% of infertile males. Some genetic abnormalities associated with male infertility consist of chromosomal disorders such as Klinefelter’s syndrome [23,24,25], cryptorchidism [26,27], Y chromosome microdeletions [28,29] and defective androgen synthesis or response [30,31]. The acquired conditions of primary infertility comprehend a wide group of factors negatively affecting fertility, including environmental [32,33] and lifestyle factors, such as smoking [34], alcohol [35] and drug abuse [36], infections [37] and obesity [38]. Furthermore, other important acquired conditions involve the insurgence of testicular cancer [39] and vascular abnormalities, such as varicocele, which affect spermatogenesis [40,41].

Pathological conditions related to the pretesticular causes of infertility affect about 1–2% of infertile males and are mainly due to gonadotropin-releasing hormone, luteinizing hormone and follicle-stimulating hormone deficiency. These conditions are generally provoked by hypothalamic–pituitary diseases, such as Kallmann and Prader-Willi syndromes, systematic disorders and pituitary and hypothalamic tumors [42,43,44].

In about 30–40% of cases, infertile males do not clearly fall within any of the aforementioned three groups, and they are generally classified as idiopathic [13,14,45,46], i.e., the reason for the infertility condition is very presumably destined to be undiagnosed.

### 1.2. Laboratory Routine Diagnosis of Male Infertility

In 1980, the World Health Organization (WHO) defined the first guidelines and the criteria to perform a correct semen analysis, indicating the proper laboratory methods and reference values for the examination of the sperm. The last updating was published in 2010 and introduced many significant changes [47]. Sperm analysis is the central laboratory examination indicated by WHO as the diagnostic path to approach a case of male infertility. The parameters considered in the evaluation of the seminal fluid, whose alteration is often indicative of specific diseases, are volume, pH, liquefaction, viscosity, number of spermatozoa, concentration/ejaculate (normal ≥ 15 million/ejaculate), motility, morphology and other optional parameters. The terminology used in the description of the semen analysis is normozoospermia (the outcome of the values close to normal); oligozoospermia (total number of spermatozoa below the lower reference limit); asthenozoospermia (total motility, calculated as progressive + non-progressive motility, ≤ 40% or progressive motility ≤ 32%); teratozoospermia (spermatozoa with normal morphology ≤ 4%); oligoasthenoteratozoospermia (abnormality of all three previous variables); azoospermia (absence of spermatozoa in the ejaculate); aspermia (absence of ejaculation) [47,48]. Oligozoospermia, a medical condition characterized by low sperm count and quality, is associated with 90% of male infertility cases [49,50]. Paradoxically, results of the sperm analysis do not give clinicians and patients the absolute certainty that one or more alterations in the sperm morpho-functional parameters render, beyond any doubt, a given subject unable to procreate. In fact, the ranges indicated by WHO are those found in the 95% of normal fertile males, i.e., reference values do not provide a cutoff for infertility, but rather a range of values found in the 95% of men who achieved fatherhood. This strongly confirms the need for additional laboratory analyses capable of providing biochemical/molecular information useful for both diagnosis and treatment of male infertility, rather than simple morpho-functional sperm evaluation not necessarily associated with male infertility. Since the molecular mechanisms underlying alterations in sperm analysis remain unknown, many scientific studies have focused on causal gene mutations, the role of epigenetics, post-translational modifications, sperm DNA fragmentation and the role of oxidative/nitrosative stress in men affected by idiopathic infertility [51].

## 2. Male Infertility and Oxidative/Nitrosative Stress: A Rationale for Antioxidant-Based Therapies

One of the hallmarks of most of the aforementioned conditions affecting infertile males is the evidence of oxidative stress in their seminal fluid [52,53,54,55]. This condition is defined as the imbalance between the production of reactive oxygen species (ROS) and the protective action of the antioxidant systems responsible for their neutralization and removal. This group (ROS) of harmful molecules, which have a half-life of nanoseconds, include singlet oxygen (^1^O_2_), superoxide anion (O_2_^●−^) and hydroxyl radical (^●^OH). The non-radical hydrogen peroxide (H_2_O_2_) is generally included within ROS due to its ability to form ^●^OH when reacting with reduced transition metals (Fenton reaction). Hydroxyl radicals may also be produced through the interaction of superoxide anions with oxidized transition metals (Haber–Weiss reaction). As well as in other biological contexts, also in sperm cells, oxidative stress is associated with nitrosative stress. This is defined as an uncontrolled production of reactive nitrogen species (RNS), mainly deriving by the spontaneous decomposition of nitric oxide (^●^NO) and by its reaction with O_2_^●−^. Excess production of ^●^NO occurs through the activity of the different isoforms of nitric oxide synthase (NOS), of which the inducible isoform (iNOS) is generally involved in nitrosative stress. When ^●^NO combines with O_2_^●−^, the product of the reaction is the powerful non-radical oxidant peroxynitrite (ONOO^−^), which, compared to ROS and RNS, has a relatively long half-life at neutral pH (~5 s). Peroxynitrite and ^●^NO decompose into various unstable RNS, some of which (nitrite and nitrate) are considered the stable^. ●^NO end-products. Their quantification in biological samples is taken as an indirect measurement of nitrosative stress.

Recent data have shown that disorders such as poor fertilization, pregnancy loss, birth defects and poor embryonic development are correlated with high susceptibility of spermatozoa to oxidative insult [56,57]. Since the excess production of ROS and RNS are able to induce irreversible modifications to biologically fundamental macromolecules of spermatozoa, such as lipids, proteins and nucleic acids, it has been found that oxidative/nitrosative stress provokes defects in sperm functions by decreasing their motility and fertilizing capacity. In particular, it has been demonstrated that increased levels of ROS and RNS stimulate lipid peroxidation of polyunsaturated fatty acid-rich plasma membranes of spermatozoa [58,59,60] and DNA fragmentation [61,62,63]. Furthermore, as well as in other pathological conditions such as neurodegenerative diseases, ischemia and cancer [64,65,66,67], the insurgence of oxidative/nitrosative stress in spermatozoa is associated with mitochondrial dysfunction. The damaging activities of ROS and RNS on proteins provoke permanent modifications of their structures through thiol oxidation, sulfonylation and tyrosine nitrosylation reactions. One of the targets of the harmful action of these toxic compounds is represented by altered expressions and catalytic activities of glycolytic enzymes, Krebs’ cycle enzymes, protein complexes of the electron transfer chain (ETC) and oxidative phosphorylation (OXPHOS). Altogether, these changes of monosaccharide metabolism (glucose and fructose) of spermatozoa lead to reduced efficiency in ATP production, with a consequent energy crisis and mitochondrial dysfunction [68,69]. In this scenario, many studies, in recent years, focused on the correlation between oxidative/nitrosative stress and male infertility, identifying many promising biomarkers in the seminal fluid (derived by the damaging action of ROS and RNS overproduction on spermatozoa macromolecules) such as malondialdehyde (MDA), 4-hydroxynonenal (4-HNE), 8-hydroxy-2′-deoxyguanosine (8-OHdG), nitrites and nitrates (NO_2_^−^ and NO_3_^−^) [70,71,72,73].

**Figure 1:**
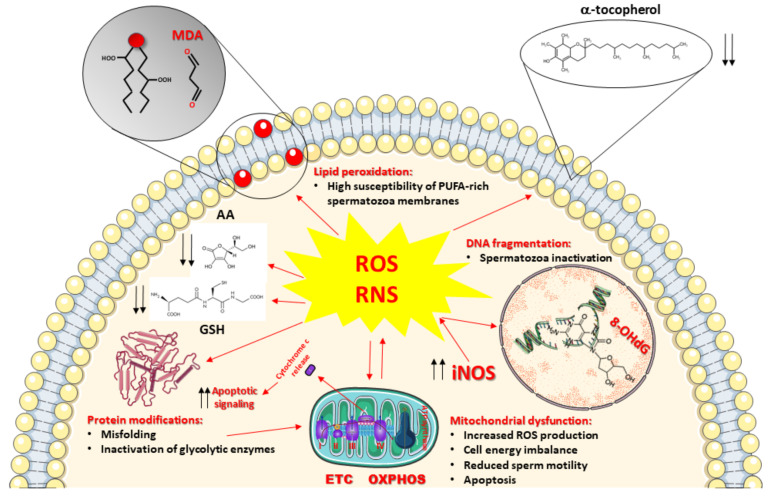
Schematic representation of the main sources and damaging effects of reactive oxygen species (ROS) and reactive nitrogen species (RNS) overproduction. Mitochondria are both the main intracellular source and one of the main targets of ROS. Incorrect transport of electrons through the electron transport chain (ETC) exacerbates mitochondrial ROS generation, causes a decrease in oxidative phosphorylation (OXPHOS) efficiency with a consequent decrease in ATP production and energy crisis. Increased expression of the inducible form of nitric oxide synthase (iNOS) is responsible for increased nitric oxide production. Its decomposition and reaction with ROS generate further RNS. Sustained oxidative/nitrosative stress provokes a decrease in intracellular levels of ascorbic acid (AA) and reduced glutathione (GSH), as well as those of the membrane-bound α-tocopherol. The decrease in low-molecular-weight antioxidant defenses allows ROS and RNS to attack the most important biomolecules, causing: (i) the induction of the lipid peroxidation reaction chain by acting on the double bonds of the polyunsaturated fatty acids (PUFAs) of membrane phospholipids and leading to the formation of various decomposition products including malondialdehyde (MDA); (ii) the increase in protein misfolding and unfolding with loss of the protein biological activities, including the activities of glycolytic enzymes that further imbalance mitochondrial functioning and energy metabolism; (iii) the oxidative damage to DNA, causing its fragmentation and generation of DNA oxidative products such as 8-hydroxy-2′-deoxyguanosine (8-OHdG). Mitochondrial malfunctioning leads to loss of cytochrome c from the intermembrane space into the cytoplasm, acting as a signal for triggering apoptosis. Decreased morpho-functional parameters of spermatozoa, deeply affecting male fertility, are the ultimate consequences of ROS and RNS damaging activities.

To counteract ROS and RNS overproduction and to prevent the insurgence of oxidative/nitrosative stress, cells of all oxygen-dependent organisms are provided with a pool of ROS and RNS scavenging enzymes (SOD, catalase, glutathione peroxidase, heme oxygenase, thioredoxin), representing the enzymatic category of the antioxidant defenses of the cells and capable to efficiently remove selected ROS (O_2_^●−^, H_2_O_2_, organic peroxides). To account for wider protection aimed to eliminate other dangerous species (^●^OH, ONOO^−^), cells contain various low-molecular-weight antioxidants having, as a common characteristic, remarkable reducing power [74]. Therefore, these compounds are capable of interacting nearly with any type of oxidant, including ROS and RNS. The gross division of low-molecular-weight antioxidants into hydrophilic (water-soluble) and hydrophobic (fat-soluble) confines them into water-rich (cytoplasm, mitochondrial matrix) and fat-rich (biological membranes) cell compartments, thus discriminating (and/or limiting) their ability to scavenge ROS and RNS. Figure 1 summarizes ROS and RNS damaging activities and the location of the main water- and fat-soluble low-molecular-weight antioxidants within cells.

### 2.1. ROS and RNS from Non-Seminal Cells

Oxidative/nitrosative stress may originate in cells different from spermatozoa, ultimately causing an accumulation in seminal plasma of various biomarkers deriving from the damaging activities of these toxic compounds. Various studies have demonstrated the pathobiological association between varicocele and increased oxidative/nitrosative stress. Varicocele affects about 30–40% of infertile males [40] and is defined as an abnormal dilation of the pampiniform venous plexus in the scrotum that alters hemodynamics and testicular microenvironment, such as increased scrotal temperature, causing defects in spermatogenesis. Notwithstanding that an imbalance between ROS and RNS production and antioxidant defenses has been found in the semen of patients with varicocele [75,76,77], the definite correlation between oxidative/nitrosative stress and varicocele-induced infertility has not been completely clarified. Increased production of proinflammatory cytokines, such as the interleukins (IL) IL-1 and IL-6 [78], activation of iNOS with the consequent increase in ^●^NO production and alteration in testicular arterial blood flow [79], heat-induced mitochondrial dysfunction [80] and coenzyme Q_10_ deficiency [81] have been proposed as valuable evidence connecting increased ROS and RNS production with infertility in patients with varicocele.

Increased production of ROS and RNS may also be caused by infections of the male reproductive tract that lead to both unspecific and specific immune responses, inducing inflammation, tissue infiltrations of leukocytes and leukocytospermia during the second and the third stage of infections [82]. However, since low levels of leukocytes are present even in ejaculates of healthy subjects, WHO has defined the condition of leukocytospermia as the presence of these cells in the ejaculate above the threshold value > 1 × 10^6^ spermatozoa/mL [47]. An early and effective mechanism against infections is represented by the killing of microbes via the oxidative burst of polymorphonuclear leukocytes and macrophages. These cells represent the main producers of ROS in the male genital tract [83], and their contribution to the insurgence of oxidative stress is mainly related to infections of epididymis or testes since the presence of ROS produced by leukocytes in the ejaculate is short-lived. Nevertheless, the pathobiological role of leukocytospermia in the excessive production of ROS and in its correlation with male infertility is still under debate. In effect, several studies have demonstrated that this parameter is strictly connected with damages to spermatozoa components, such as membranes and DNA, even when the level of leukocytospermia is below the threshold value established by WHO [84,85,86].

An increase in oxidative/nitrosative stress is a common feature of many different types of cancer, including testicular cancer [87,88]. It is known that malignant cells induce alterations in the testicular microenvironment, causing oxidative imbalance and defects in sperm functions [89,90]. Other important sources of ROS and RNS derive from factors related to unhealthy lifestyles, such as smoking, excessive alcohol intake and drug abuse [91]. The presence of nicotine and other carcinogenic chemical products in tobacco smoke is able to greatly increase the production of ROS and RNS coupled to the decrease of antioxidants in semen, leading to DNA fragmentation and lipid peroxidation of spermatozoa [92,93]. Furthermore, prolonged exposure to temperatures higher than 37 °C, as well as to many environmental xenobiotics such as air pollutants, heavy metals (cadmium, lead, nickel, iron and copper), ionizing and nonionizing radiations, can contribute to the insurgence of oxidative/nitrosative stress.

### 2.2. ROS and RNS in Sperm Cells: The ROS Paradox and the Sperm Antioxidant Defenses

As for the other cell types of aerobic organisms, spermatozoa rely on oxidative phosphorylation (OXPHOS) as the major source of energy production in terms of ATP molecules. It has been demonstrated that OXPHOS mainly occurs in the sperm mid-piece, a mitochondrial rich district, differently from glycolysis that takes place in the head and in the fibrous sheath of the flagellum, where glycolytic enzymes are anchored [94]. Mitochondrial metabolism represents one of the major endogenous sources of ROS deriving from the activity of the electron transport chain (ETC) complexes. The electron flux through ETC and the concomitant transport of H^+^ ions in the intermembrane space of mitochondria performed by complexes I, III and IV are utilized to catalyze the ATP synthesis and the tetravalent reduction of O_2_ to H_2_O. Despite this process is extremely efficient and finely regulated, a considerable amount of superoxide and hydroxyl radicals are physiologically produced (representing about 1% of total oxygen consumed by the cell), in particular by complexes I and III [95,96].

Another important endogenous source of ROS and RNS in semen is represented by immature spermatozoa deriving from defective stages of spermatogenesis. During the last phase of gamete differentiation (the spermiogenesis stage that occurs in the testis), spermatids undergo the maturation process, characterized by nucleus remodeling, chromatin condensation, acrosome and sperm tail formation and excess cytoplasm removal (the maturation stages occurring in the epididymis) [97]. Defects in this latter process give rise to immature spermatozoa, marked by retained cytoplasmic droplets in the mid-piece [98], containing glucose-6-phosphate dehydrogenase (G6PDH) enzyme. G6PDH is a cytoplasmic enzyme involved in the first reaction of the pentose phosphate pathway (PPP). It uses oxidized nicotinamide adenine dinucleotide phosphate (NADP^+^) to oxidize glucose-6-phosphate to 6-phosphogluconate, thus generating reduced nicotinamide adenine dinucleotide phosphate (NADPH). Notwithstanding the maintenance of adequate levels of the reduced form of glutathione (GSH) needs high NADPH levels to ensure the correct activity of glutathione reductase, it has been demonstrated that high NADPH concentrations favor ROS production in immature spermatozoa through the activity of the Ca^++^-dependent, membrane-bound isoform 5 of NADPH oxidase (NOX5) [99,100,101]. Additional endogenous ROS production is obtained, even if in modest contribution, from peroxisomal β-oxidation, the catabolic pathway of very long fatty acids and from the activity of the enzymes of the cytochrome P_450_ family involved in xenobiotic detoxification [96,102].

**Figure 2:**
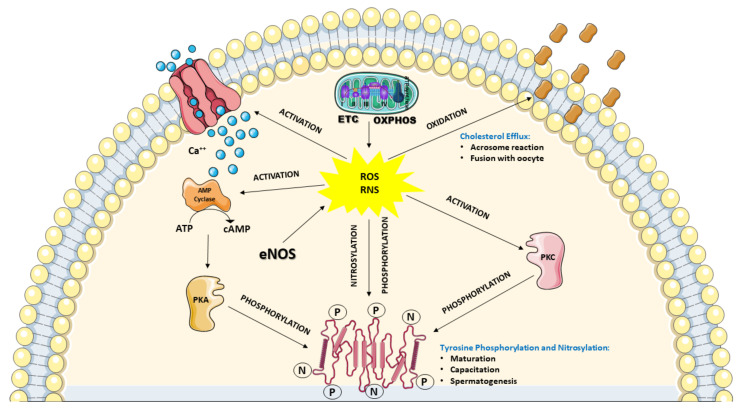
Schematic representation of the involvement of ROS and RNS to ensure the correct development and function of spermatozoa. In functional mitochondria, the correct coupling between ETC and OXPHOS ensures an adequate ATP supply for cell energy demand, as well as a “physiologic” flux of ROS (mainly in the form of superoxide anion). At the same time, the endothelial (and possibly the neuronal) nitric oxide synthase isoform (eNOS) provides “physiologic” production of RNS (in the form of nitric oxide). These controlled amounts of ROS and RNS positively affects numerous intracellular processes of spermatozoa, including; (i) influx of calcium ions (Ca^++^) triggering the cyclic AMP (cAMP) cascade (increase in adenylate cyclase activity) leading to activation of protein kinase A (PKA) which catalyzes the phosphorylation of numerous target proteins; (ii) the concomitant activation of protein kinase C (PKC), producing a further increase in protein phosphorylation; (iii) regulation of protein activities through nitrosylation reactions; (iiii) oxidation of cholesterol and removal from the plasma membrane. Some of these processes are deeply involved during spermatogenesis, maturation and capacitation, as well as in acrosome reaction and fusion with the oocyte. Due to these functions in their life cycle, spermatozoa may be defined as “professional generators” of ROS and RNS. During these ROS and RNS-mediated processes, no significant depletion in the antioxidant heritage of spermatozoa occurs.

The above-depicted scenario highlights the evidence that a considerable amount of ROS and RNS are physiologically produced by the spermatozoa themselves, identifying these cells as “professional generators of ROS”. Notwithstanding the aforementioned destructive effects that ROS and RNS can induce on sperm functions, many studies clearly demonstrated that controlled levels of ROS and RNS are necessary for spermatozoa to acquire their fertilization ability during the differentiation in the testis, the maturation in epididymis and the capacitation in the female reproductive tract. In particular, it has been shown that moderate H_2_O_2_ levels are required for correct spermatogenesis in the testis. Indeed, H_2_O_2_ regulates the stem cell population and the progenitor cells from which spermatozoa originate, the chromatin condensation in the nucleus and the mitochondria arrangement in the mid-piece of spermatozoa. This type of regulation is mainly performed by promoting the subsequent phosphorylation of different tyrosine and serine amino acidic residues of various target proteins [103,104], and it is also required for the maturation process occurring in the epididymis, in which spermatozoa undergo DNA compaction and acquisition of motility [89]. Furthermore, much evidence has demonstrated that moderate levels of ROS and RNS are necessary for the correct development of capacitation, again performed through the redox-modulated tyrosine phosphorylation of target proteins. In particular, H_2_O_2_, ^●^NO, and O_2_^●−^ levels regulate the phosphorylation of two tyrosine-rich proteins of 81 and 105 kDa, which are selectively overexpressed during capacitation. The post-translational modification of these proteins, occurring through the concomitant activation of tyrosine kinase and protein kinase C and inactivation of phosphatases, is fundamental to promote the subsequent oxidation of specific cysteine residues necessary for their ultimate activation [105]. Moreover, ROS, in conjunction with the intracellular Ca^++^ entry via the cation channels of sperm CatSper, are able to stimulate the increase in intracellular cyclic AMP (cAMP) levels that leads to the activation of protein kinase A (PKA), indirectly increasing the tyrosine phosphorylation of proteins [105]. This latter mechanism, again regulated by H_2_O_2_, ^●^NO, and O_2_^●−^, is also extended to proteins located in the flagellar fibrous sheath involved in hyperactivation (i.e., the acquisition by spermatozoa of nonlinear motility) and to proteins involved in acrosome reaction and sperm-oocyte fusion [106,107]. In order to acquire the ability to perform the acrosome reaction and fusion with the oocyte, spermatozoa need to undergo modifications of their plasma membrane composition. In particular, it has been shown that ROS are necessary to stimulate cholesterol oxidation and its consequent removal from the plasma membrane. This process is accompanied by the activation of phospholipase A2 leading to an increase in polyunsaturated fatty acids cleavage, ultimately causing an increase in plasma membrane fluidity [108,109]. Figure 2 summarizes ROS and RNS activities that are considered fundamental for the correct development and function of spermatozoa.

In consideration of the abovementioned fundamental beneficial role of ROS and RNS in the differentiation and the acquisition of the fertilization ability by spermatozoa on one side, but on the other hand of their harmful role in the insurgence of oxidative/nitrosative stress, seminal plasma has evolved as one of the most powerful antioxidant-rich fluids in human beings, in order to finely control the amount of ROS and RNS. Despite spermatozoa are equipped with endogenous antioxidant defenses against oxidative/nitrosative stress, for obvious structural reasons, these cells are highly dependent on the powerful antioxidant properties of the fluids that surround them, i.e., the seminal plasma released from accessory glands, following spermatozoa release from the germinal epithelium. The antioxidant profile, characteristic of this body fluid, comprehends both enzymatic and nonenzymatic antioxidants that are able to detoxify ROS and RNS, avoiding the possible insurgence of oxidative/nitrosative stress. The enzymatic scavengers include catalase, superoxide dismutase (SOD), glutathione peroxidase (GPx) and glutathione-S-transferase (GST) [110,111,112]. The nonenzymatic antioxidant profile of seminal plasma is represented by a heterogeneous group of both endogenous and exogenous low-molecular-weight scavengers divided into water-soluble (such as ascorbic acid, GSH, uric acid, polyphenols) and fat-soluble compounds (such as α-tocopherol, γ-tocopherol, all-trans-retinoic acid, all-trans-retinol, carotenoids and coenzyme Q_10_) [113,114,115]. Giving the importance of maintaining adequate levels of each component of this set of antioxidants to ensure the wellness of spermatozoa and, therefore, their intrinsic fertilizing capacity, it is a very common clinical practice to indiscriminately prescribe to infertile males repeated cycles of administration of various types of nutraceutical/drug-containing antioxidant to improve their fertilization capacity. This therapeutic approach is generally adopted without previously screened patients to determine the concentration of antioxidants in seminal plasma, i.e., antioxidant treatment is very often administered without knowing its real need caused by a deficiency in one or more of the low-molecular-weight antioxidants.

With the aim to understand the actual effectiveness of antioxidant-based therapies in the treatment of male infertility, in this review, we analyzed data from the literature concerning the use of low-molecular-weight antioxidants to ameliorate the sperm quality and the spermatozoa fertilization ability of patients affected by different conditions of infertility. We limited the evaluation of the literature (using PubMed as the data bank for consultation, using all the different mix of the search words, antioxidants, male infertility, low molecular-weight antioxidants, humans, sperm, spermatozoa) by considering only clinical studies performed during the last ten years and focusing on those in which compounds having a direct and well-established antioxidant activity towards ROS and/or RNS were used.

## 3. Antioxidant-Based Therapies in the Treatment of Male Infertility

### 3.1. Ascorbic Acid (Vitamin C)

Ascorbic acid (AA), also known as vitamin C, is one of the most abundant water-soluble antioxidants within mammalian tissues, acting as a reducing cofactor in several enzymatic reactions [116]. AA is an essential cellular antioxidant also in conditions of abundance of GSH, protecting tissues, organs and cells from the harmful effects of ROS and RNS overproduction during oxidative/nitrosative stress [117]. The standard redox potential of the couple oxidized/reduced ascorbate (+0.08 V at pH 7.0) qualifies AA as a fairly good reducing agent, capable to quickly react with a wide range of ROS and RNS, including superoxide, peroxynitrite and hydroxyl radicals, through one or two electrons reactions generating, respectively, ascorbyl radical or dehydroascorbic acid (DHA). DHA can be reconverted back to the reduced AA form in a direct chemical reaction involving glutathione (GSH), even though NADPH-dependent enzymatic reactions involving glutaredoxin, thioredoxin and GSH reductases have also been demonstrated. The antioxidant protection performed by AA is also related to the recycling of oxidized α-tocopherol (vitamin E), in particular, contributing to the maintenance of the α-tocopherol redox state within biological membranes. Since AA is not synthesized by most mammals (including human beings), the human body depends on vitamin C-rich food ingestion to satisfy its AA need [118], with a recommended daily intake (RDI) of 65 to 90 mg/day. The inability to synthesize AA is due to the loss of function of the biosynthetic enzyme L-gulono-1,4-lactone oxidase [119]. Two distinct transporter families are involved in the cellular absorption of AA, depending on the oxidative state of the molecule. Indeed, facilitate DHA internalization is performed through the hexose transporters (GLUT1, GLUT3 and GLUT4). Differently, AA is transported by high-affinity, low-capacity active sodium-ascorbate co-transporters (SVCT1 and SVCT2) [120]. Notwithstanding that circulating levels of AA are relatively low (30 to 70 µmol/L in the serum of healthy humans) [113], concentrations of this important antioxidant in peripheral tissues are, interestingly, elevated. Particularly in the brain, AA is highly concentrated, usually reaching concentrations up to 3000 µmol/L brain water [121]. In the testis, AA levels hover around 3 mg/100 g wet tissue (about 140 µmol/L tissue water) [122]. Expression of SCVT2 and some isoforms of the GLUT transporters have been found in Sertoli cells that form the blood-testis barrier via tight and adherens junctions. These cells are able to regulate AA concentrations in the seminiferous epithelium, underlying the key role of AA in the spermatogenesis [123]. In seminal plasma, concentrations of AA have been found up to 10 times higher than those measured in plasma, with values ranging from 200 to 350 µmol/L. Explanations of the mechanisms allowing to reach such abundant vitamin C levels in this peculiar body fluid are still unclear [113,124]. In general, since AA concentrations are higher in peripheral tissues than in plasma, the cellular internalization of AA via SVCT1 and SVCT2 occurs against a concentration gradient; the energy to overcome the electrochemical barrier is provided by the favorable inward gradient for Na^+^, which is fostered by the regular extrusion of Na^+^ via the Na^+^/K^+^-ATPase pump. The electrogenic transport is exploited by SVCT1 only, since the SVCT2 transport mechanism also involves Ca^2+^ and Mg^2+^. As mentioned above, AA is the most abundant water-soluble low-molecular-weight antioxidants in the seminal plasma [70,113,124], playing significant protection against oxidative damage to DNA [125] and prevention of semen agglutination [126]. Furthermore, AA levels in seminal plasma positively correlate not only with normal semen parameters, such as sperm volume, sperm count and sperm motility [127] but also with GSH concentration and SOD activity, suggesting a close relationship between different antioxidant defensive mechanisms against ROS-mediated damages [128,129]. Under pathological conditions leading to male infertility, in which the insurgence of oxidative stress is involved, it was observed an overall depletion of AA in the seminal fluid due to the sustained presence of ROS and RNS. In particular, in patients affected by varicocele of various grade, by genitourinary infections and by idiopathic infertility, a net decrease in AA concentration in the seminal fluid has been demonstrated [70,128,130,131].

Several studies focused on the association between AA dietary intake and semen quality. In a cross-sectional study conducted in 210 young, healthy subjects, ranging between 18 and 23 years of age, Mínguez-Alarcón L et al. [132] reported a positive correlation between the dietary intake of several antioxidant nutrients (β-cryptoxanthin, vitamin C, lycopene and β-carotene) and the total motile sperm count, underlying that an AA intake satisfying the RDI was positively associated with increased semen volume. In a case–control study conducted on 31 normozoospermic subjects and 30 infertile males affected by moderate or severe oligozoospermia and severe teratozoospermia, it has been shown that low intake of antioxidant nutrients seems to worsen semen quality while the regular ingestion of vitamin C-reach food improves the morpho-functional characteristics of the semen [133]. Furthermore, in a study realized on 107 asthenozoospermic patients and 235 age-matched controls, Eslamian et al. reported a significant negative relationship between a dietary pattern of antioxidant-rich foods (including vitamin E, vitamin D, vitamin C, zinc, folate, total fiber, selenium and PUFAs) and risks of asthenozoospermia in a cohort of Iranian men [134].

The majority of the clinical studies aimed to evaluate the effect of vitamin C supplementation on male infertility were carried out by applying the simultaneous administration of various antioxidants, including AA. The administration of AA only was conducted in three clinical trials, in which different semen parameters and fertilization capacity of infertile males were evaluated. When examining the effect of AA supplementation on parameters of the sperm analysis, results reported in the available studies are contradictory. In a controlled clinical trial, 115 infertile males affected by varicocele and subjected to varicocelectomy were divided into two groups receiving, twice a day, either 250 mg of AA (AA group) or placebo (control group) for the next three months after surgery. The AA group showed no increase in sperm count and significant amelioration of sperm motility and morphology, suggesting that AA administration positively affected only quality but not the number of spermatozoa [135]. Differently, Rafiee et al. [136] reported that the administration of a single dose of 1000 mg/day to 200 subjects with increasing body mass index and ages ranging from 20 to 60 year was able to improve the sperm concentration and motility, but not the semen volume and the percentage of normal spermatozoa morphology. In a cross-sectional study carried out in 120 male workers, occupationally exposed to lead and to show testicular dysfunction and high rate of DNA fragmentation, it was found that the administration of 1000 mg/day of AA for five consecutive days/week for up to 3 months, statistically increased sperm motility, sperm total count and significantly decreased the number of morphologically abnormal spermatozoa [137]. These results prompted the authors to suggest that AA treatment could offer protection against sperm damage caused by a lead-mediated increase of oxidative stress [137].

As previously said, in most studies, exogenous AA was administered in combination with other antioxidants (such as vitamin E, carotenoids and coenzyme Q_10_), thus rendering it difficult to extrapolate the potential vitamin C beneficial effects on male patients affected by infertility. In a study conducted on 20 infertile males with known asthenoteratozoospermia, the combined daily supplementation with AA (60 mg), L-carnitine (1500 mg), coenzyme Q_10_ (20 mg), vitamin E (10 mg), vitamin B_9_ (200 µg), vitamin B_12_ (1 µg), zinc (10 mg) and selenium (50 µg) was able to induce, after 3 months of the administration, a statistical improvement of all semen parameters and DNA integrity. Although a remarkable variability in the inter-patient response to treatment was also shown, the authors reported a pregnancy rate of 5% [138]. Interestingly, the same administration protocol was applied to 20 males affected by grade I varicocele resulting in a significant reduction of DNA fragmentation and highly degraded sperm cells, a significant increase in total sperm cells, but no effects on other semen parameters [139]. In 210 infertile males affected by severe oligoasthenoteratozoospermia, Magdi et al. [140] tested the effects of the daily administration of AA (1 g), vitamin E (400 mg) and L-carnitine (2 g) as a possible management protocol of clinical utility. After six months of treatment, a significant increase in sperm concentration, total sperm count, percentage of spermatozoa with total motility and progressive motility, as well as a significant reduction of spermatozoa with abnormal morphology, was observed [140]. It should, however, be underlined that the real effects of this antioxidant protocol were confounded by the change in patient lifestyle adopted in the study protocol and aimed at minimizing adverse lifestyle factors [140]. Differently, the daily supplementation with 80 mg of AA, 120 mg of coenzyme Q_10_ and 40 mg of vitamin E was reported to be ineffective, after 3 and 6 months of treatment, in changing both atypical sperm cell number and semen volume, but capable of inducing significant improvements in sperm cell concentration and sperm motility in a cohort of 169 males affected by idiopathic oligoasthenoteratozoospermia [141]. Worthy of note is that after six months, a total of 48 pregnancies, comprising 16 spontaneous pregnancies (i.e., pregnancies occurring with no need of any type of ART) and 32 pregnancies requiring ART, were achieved by this group of patients using this treatment protocol [141]. In a study conducted on 15 infertile patients with oligozoospermia and/or asthenozoospermia, Terai et al. [142] reported that the combined administration of 1000 mg of AA, 750.1 mg of L-carnitine, 30 mg of zinc, 16.05 mg of astaxanthin, 90.26 mg of coenzyme Q_10_, 60.1 µg of vitamin B_12_ and 150 mg of vitamin E three times a day for 12 weeks, significantly improved only total motile sperm count, with no effects on the other semen parameters. In a single-blinded clinical trial, Sadaghiani et al. [143] reported that the daily administration for three consecutive months of 100 mg of AA, 30 mg of coenzyme Q_10_, 8 mg of zinc, 12 mg of vitamin E and 400 μg of folic acid, combined with that of 200 mg of selenium every other day, to 50 infertile oligozoospermic and asthenozoospermic male smokers, beneficially affected various morpho-functional semen parameters (concentration, pH, volume, total motility, morphology, count and progressive motility). A substantially different scenario was depicted by Steiner et al. [144], who conducted a randomized controlled trial on 174 infertile males (categorized into oligozoospermic, asthenozoospermic, teratozoospermic and DNA fragmented patients), analyzed for sperm analysis anomalies and clinical outcome measures of fertility (pregnancy and live birth rates). In this study, infertile males were randomly assigned to a group receiving placebo and a group receiving the daily administration of an antioxidant formulation composed of 500 mg of AA, 400 mg of vitamin E, 0.20 mg of selenium, 1000 mg of L-carnitine, 20 mg of zinc, 1000 mg of folic acid, 10 mg of lycopene, and 2000 IU of vitamin D, for at least 3 months and up to 6 months. The authors reported that, compared to the placebo group, this antioxidant formulation and administration protocol did not result in any statistically significant improvement of the sperm analysis. Even more important, these results suggested that antioxidant treatment is not able to improve in vivo pregnancy or live-birth rates of couples with male factor infertility. A summary of the clinical studies concerning the effects of AA on male infertility is provided in Table 1.

### 3.2. N-Acetylcysteine (NAC)

GSH is the other most abundant hydrophilic low-molecular-weight antioxidant that, differently from AA, mammals are able to synthesize [145]. It is a tripeptide, γ-L-glutamyl-L-cysteinyl glycine, present in concentrations ranging between 1 and 10 mM in animal tissues. GSH is synthesized via a two-step ATP-requiring enzymatic reactions; the first step is catalyzed by glutamate-cysteine ligase (GCL) that conjugates cysteine with glutamate, producing γ-glutamylcysteine. The second step is catalyzed by GSH synthase, which adds glycine to γ-glutamylcysteine producing the final product, GSH. This hydrophilic compound exists in the thiol-reduced (GSH) and disulfide-oxidized (GSSG) forms [146]. GSH is the most abundant of the two species in eukaryotic cells (the GSH/GSSG ratio is about 100), mainly localized in cytoplasm and mitochondria, and having different concentrations in the various tissues. Notwithstanding that GSH is involved in the modulation of cell proliferation, apoptosis, inflammatory processes, redox signaling and detoxification of xenobiotics, probably the main function of this hydrophilic compound is to act as an antioxidant [145]. In particular, thanks to its sulfhydryl group (-SH) that confers active protection against ROS, GSH is involved in a redox cycle for the detoxification of hydrogen peroxide and lipid peroxides. These potentially dangerous compounds are reduced through the activity of the two selenium-dependent and selenium-independent forms of the enzyme GPx, in a reaction that oxidizes two GSH molecules into one GSSG molecule [147]. The regeneration of GSH is accomplished by glutathione reductase (GR), responsible for the maintenance of high values of the GSH/GSSG ratio, in the presence of reduced NADPH. Hence, GSH homeostasis is strictly connected to the PPP and the malic enzyme activity, i.e., the two main cellular sites for NADPH generation [148]. In addition to the involvement in the aforementioned redox cycle, GSH also acts as the cofactor of the enzyme family of GSTs. These protective enzymes are a large group of cytosolic and membrane-bound isoenzymes involved in the detoxification of xenobiotics (potentially toxic compounds often causing an increase in oxidative/nitrosative stress), obtained through the conjugation of GSH to xenobiotic substrates for their faster extrusion from the cell. In sperm cells, GSTs are involved not only in preventing lipid peroxidation in conditions of high levels of ROS and RNS but also in cell signaling regulation during spermatogenesis, sperm capacitation and fertilization [149].

Since the -SH group of GSH is supplied by cysteine, high levels of this amino acid are essential for GSH biosynthesis, therefore ensuring adequate GSH tissue levels. Cysteine normally derives from the diet, from protein breakdown and from the trans-sulfuration of methionine. Due to the fundamental role of GSH in detoxifying reactions, both to scavenge ROS and RNS and to eliminate xenobiotics, increased cysteine availability is needed under conditions of oxidative/nitrosative stress. As in the case of AA, GSH levels in human tissues are remarkably higher (2500–4000 µmol/L tissue water) than its circulating concentrations ranging between 15 and 20 µmol/L [113,150]. Seminal plasma values of GSH have been found equal or higher to those present in serum, indicating a free distribution of this molecule regulated on its relative concentrations in the two fluids [113,151]. Interestingly, in pathological conditions related to male infertility, characterized by sustained oxidative/nitrosative stress, data on variations of GSH levels are somehow controversial. Indeed, in viable spermatozoa of infertile patients, Moretti et al. [152] showed increased intracellular levels of total GSH (GSH + GSSG) with respect to values detected in spermatozoa of control fertile males. The possible explanation suggested by the authors is that the higher levels of ROS and RNS in spermatozoa of the infertile group increased the demand for antioxidants to counteract oxidative/nitrosative stress, thus leading to increasing values of GSH + GSSG. This hypothesized mechanism might have been accomplished by an augmented transport of GSH into the cell through membrane transporters located on the sperm surface [152]. These results were corroborated by those reported in a study by Kralikova et al. [153], who demonstrated significantly higher concentrations of homocysteine, cysteine, cysteinyl-glycine and GSH in spermatozoa of patients with pathological sperm parameters compared to those found in normozoospermic fertile subjects. The authors reported the same aforementioned explanation for this phenomenon, i.e., the overproduction of ROS and RNS could cause an upregulation of thiol synthesis in order to protect spermatozoa against oxidative damages [153].

As said, diametrically opposite results reported an overall depletion of GSH in seminal plasma of infertile males, caused by increased oxidative/nitrosative stress. In a study conducted on 74 infertile patients affected by varicocele, genitourinary infections or idiopathic infertility, Micheli et al. [154] reported a significant concomitant decrease of GSH and AA only in seminal plasma of the varicocele group compared to the control group of fertile males. The non-significant reduction of GSH in the two other conditions of infertility was explained by the authors by the sparing effect induced by AA. Compared to a control group of 17 fertile males, Moretti et al. reported a net decrease of the GSH/GSSG ratio, in a cohort of 36 infertile patients affected by leuokocytospermia or varicocele, independently on the pathology causing infertility, even though the most striking differences were recorded in the varicocele group [155].

The aforementioned results suggest that drugs aimed to increase seminal plasma/spermatozoa levels of GSH may be successful for the treatment of male infertility. Since the oral administration of GSH is useless because the molecule is degraded before its absorption, pharmacological strategies are aimed to stimulate GSH biosynthesis to obtain a net increase of intracellular GSH concentrations. In this light, to improve the availability of cysteine, ultimately enhancing GSH synthesis, *N*-acetylcysteine (NAC) has widely been used as a potential treatment for male infertility (administered alone or in combination with other antioxidants), also thanks to its relevant properties to act as a direct ROS and RNS scavenger. In a randomized, blinded clinical trial performed by Jannatifar et al. [156], 50 infertile males with asthenoteratozoospermia received NAC orally for 3 months (600 mg/day), and their semen parameters and DNA fragmentation index were compared with those determined before the beginning of NAC treatment. The authors reported that the three months supplementation with NAC significantly improved sperm analysis parameters (count, motility and normal morphology) and significantly reduced DNA fragmentation, indicating that NAC supplementation is able to protect morpho-functional spermatozoa characteristics and to reduce ROS and RNS-mediated damages to DNA. The protective antioxidant effect of NAC against DNA damages was also reported by Barekat et al. [157], who conducted a study on 35 infertile men with varicocele. Following microsurgery, patients were randomly divided into control or treatment groups, with the former receiving no drug after varicocelectomy (*n* = 20) and the latter (*n* = 15) receiving the daily administration of NAC (200 mg/day) post-varicocelectomy for three months. In addition to the beneficial effects due to the microsurgery, the authors showed that, while control patients had a pregnancy rate of 10%, this value in patients receiving NAC supplementation increased significantly, up to 33.4%. According to these data, the authors suggested that administration of NAC following varicocelectomy may represent an added value not only for the improvement of sperm motility, a decrease of oxidative/nitrosative stress, recovery of GSH levels, protection of the DNA integrity, but also for the overall improvement of patient’s fertilization capacity [157].

The potential benefit of NAC administration to infertile males was also reported when patients received NAC in combination with other compounds (mainly antioxidants). Dattilo et al. [158] conducted an uncontrolled study on 84 male partners of couples with at least 2 previous in vitro fertilization (IVF) failures, with no evidence of organic causes of infertility and with either measurable DNA fragmentation index (DFI) or nuclear decondensation index (SDI). Patients were administered with a single daily dose (for an average period of 130 days) of a tablet containing a proprietary extract of opuntia fig fruits (100 mg) delivering tailored amounts of quercetin (0.05 mg) and betalain (0.001 mg), plus a mix of vitamins B_2_ (1.4 mg), B_3_ (16 mg), B_6_ (1.4 mg), B_9_ (400 μg), B_12_ (2.5 μg), zinc (12.5 mg), NAC (250 mg) and vitamin E (12 mg). The aim of the authors was to facilitate the endogenous production of GSH by increasing the rate of the so-called one-carbon cycle, a pathway that involves the trans-sulfuration of homocysteine favoring the de novo GSH biosynthesis. The authors reported a significant decrease of both DFI and SDI, but no modifications of the total sperm count, fast motility and normal morphology rates after the administration protocol. Notwithstanding, high clinical pregnancy rates, with 18 live births from 18 spontaneous pregnancy (i.e., pregnancy occurring with no need of any type of ART) and 15 live births from 22 initial clinical ART pregnancies (taking place at the second IVF attempt), were reported to occur in this cohort of patients [158]. Conversely, in a study conducted on 20 patients affected by β-thalassemia major, a condition characterized by hypogonadotropic hypogonadism mediated by iron deposition and generation of ROS with a consequent increase in damaged spermatozoa, a combined treatment with L-carnitine and NAC was ineffective in the improvement of fertility [159]. In this regard, the study showed that after six months of treatment (2 g/day of L-carnitine and 600 mg/day of NAC), patients had a significant increase in the SDI and teratozoospermia index. Apparently, the treatment accentuated sperm deformities, leading to the conclusion that the use of antioxidants in β-thalassemia major patients is not justified for the potential improvement of fertility [159]. A summary of the clinical studies concerning the effects of AA on male infertility is provided in Table 2.

### 3.3. Tocopherols (Vitamin E)

The term vitamin E covers eight different substances, four tocopherols (α, β, γ, δ) and four tocotrienols (α, β, γ, δ). All these fat-soluble compounds contain a chroman ring with different numbers and positions of methyl groups and a side-chain linked to position 2 of the aforementioned ring. Tocopherols contain a saturated phytyl chain, while in tocotrienols, the tail contains three isolated double bonds [160]. Due to the presence of the hydrophobic side-chain, tocopherols are mainly localized within the phospholipid bilayer of biological membranes, acting as the major lipid-soluble antioxidant that is able to block the lipid peroxidation reaction chain. This antioxidant action is exerted by the -OH group of the chromane ring that, under the condition of oxidative/nitrosative stress, donates hydrogen radical to a lipid radical or a lipid hydroperoxide radical, resulting in the termination of the lipid peroxidation reaction chain and in the formation of a relatively stable tocopheryl- or tocotrienyl-radical. These two partially oxidized forms of vitamin E will most likely depend on AA availability in the aqueous cellular compartments for their regenerations. In this oxidoreductive reaction, AA is oxidized to ascorbyl radical, subsequently undergoing either dismutation (two ascorbyl radicals), with the formation of one AA and one DHA, or to further one-electron oxidation with the generation of DHA. Notwithstanding α, β, γ and δ tocopherols have progressively decreasing ROS scavenging power [161], it has been demonstrated that γ-tocopherol is the most effective tocopherol against RNS, with particular scavenging activity towards nitrogen dioxide with which it forms the stable nitro-adduct 5-nitro-γ-tocopherol [162]. Among the eight congeners, α-tocopherol is the most abundant in the European diet and γ-tocopherol in the American diet. In general, the RDI for vitamin E is around 7–15 mg/day [163], and in adults, severe vitamin E deficiency mainly occurs as a result of genetic defects in the α-tocopherol transfer protein (α-TTP), caused by an infrequent genetic disorder known as “isolated vitamin E deficiency” or ‘ataxia with isolated vitamin E deficiency” (AVED) [164]. The most abundant tocopherol in human tissues and in blood plasma is α-tocopherol, the circulating levels of which range between 18 and 30 µmol/L [105]. The distribution to peripheral organs and tissues is achieved by the α-TTP that incorporates α-tocopherol into lipoproteins, and it is able to distinguish α-tocopherol from all incoming forms of vitamin E [165].

Due to their hydrophobicity and the lack of effective components (such as lipoproteins) that could facilitate their solubility, concentrations of tocopherols in seminal plasma, unlike AA, are about nine times lower (α-tocopherol ~3 µmol/L and γ-tocopherol ~0.06 µmol/L) than those detected in blood plasma [113], suggesting the lack of efficient carrier systems capable of accumulating these compounds in seminal plasma. Notwithstanding the low concentrations of seminal plasma tocopherols, the high levels of AA in this body fluid ensure the correct recycling of vitamin E and exploit its beneficial effect against ROS and RNS-induced lipid peroxidation. Because of their high content of polyunsaturated fatty acids, membranes of germ cells and spermatozoa are very sensitive to oxidation from high levels of ROS and RNS. Therefore, vitamin E represents for these cells one of the most effective fat-soluble antioxidants against oxidative/nitrosative stress-related damages [166].

In a case report of a patient affected by AVED, Rossato M. and Mariotti C. [164] showed normal spermatogenesis and semen parameters notwithstanding vitamin E deficiency, challenging the effective role of tocopherols in human spermatogenesis. On the other hand, several studies showed the correlation of decreased levels of tocopherols in human seminal plasma with augmented concentrations of ROS and RNS-mediated marker of lipid peroxidation (MDA) in infertile males [70,162,167,168]. In addition to the aforementioned study [126], the impact of dietary vitamin E intake was evaluated by Nadjarzadeh et al. [169] in a case control study conducted on 32 men with primary infertility due to idiopathic oligo and/or astheno- and/or teratozoospermia and 32 age-matched normal healthy donors. The authors highlighted the association between a lower intake of vitamin E, zinc and folate and a negative effect on sperm motility and morphology. Schmid et al. [170], in a cross-sectional study performed on 80 healthy fertile male volunteers (aged 22–80 years), showed that a higher dietary intake of vitamin E, AA, folate, and zinc positively correlated with lower damages to sperm DNA. Furthermore, the authors reported that older men who consumed higher levels of the aforementioned micronutrients had similar levels of sperm DNA damage than those found in young men, underlying the age-independent importance of the intake of adequate levels of antioxidants to counteract oxidative-mediated damages to sperm DNA [170]. Similarly, Salas-Huetos et al. [171] conducted a study in healthy individuals to evaluate the effect of chronic consumption of foods rich in monounsaturated fatty acids, polyunsaturated fatty acids (including α-linolenic acid, and total ω-3), magnesium and vitamin E on semen parameters related to male fertility. To this end, 50% of the volunteers fed for 14 weeks the usual Western-style diet enriched with 60 g of a mixture of nuts/day (30 g of walnuts, 15 g of almonds, and 15 g of hazelnuts) and the remaining 50% fed the usual Western-style diet avoiding nuts. The authors reported a significant improvement of the morpho-functional parameters of the sperm analysis (total sperm count, vitality, motility, and morphology) in subjects who fed a high nut diet, suggesting that these benefits may be due to a reduction in the DNA fragmentation mediated by the higher intake of the aforementioned fat-soluble antioxidants [171].

The effectiveness of the administration of vitamin E only in the treatment of male infertility is, unfortunately, particularly limited. In this regard, Ener et al. [172] conducted a study on 45 varicocele-affected patients who underwent sub-inguinal varicocelectomy. After surgery, patients were randomized into two groups, one of which (*n* = 22) received daily oral vitamin E capsules (2 × 300 mg) for 12 months, while the other (*n* = 23) served as the control group and did not receive any supplementation. Besides the positive effects caused by the operational procedure to remove varicocele (increase in both total sperm count and motile spermatozoa), compared to the control group, the authors failed to show any additional amelioration attributable to vitamin E administration, including the pregnancy rate [172]. In a study conducted on 134 subjects, no differences in seminal plasma levels of α-tocopherols in the different groups of participants, divided according to their sperm analysis alterations, were found. However, the authors reported significantly decreased concentrations of γ-tocopherol only in the group of varicocele patients, underlying the importance of this isoform in a detoxifying varicocele-related increase of RNS [162]. Additionally, no significant differences in any parameter of the sperm analysis after the supplementation of 800 IU of α-tocopherol for 3 months to ten infertile patients (selected from a larger cohort of 134 sperm donors, including infertile patients with both sperm analysis anomalies and varicocele) were observed. However, the authors showed an increase in blood α-tocopherol after 45 days (+88%), mirrored by a decrease in γ-tocopherol (−33%) after 45 days of α-tocopherol supplementation. At the same time point, semen α-tocopherol increased by 38%, while no changes were found in γ-tocopherol concentrations. The authors speculated that decrease in γ-tocopherol levels after α-tocopherol supplementation could be due to the hepatic α-TTP, which preferentially incorporates α-tocopherol into the plasma, as well as increasing hepatic catabolism of γ-tocopherol. Moreover, authors suggested that the decrease in γ-tocopherol levels after α-tocopherol supplementation could be potentially deleterious and that oral supplements of vitamin E should contain a combination of both tocopherols, to avoid the replacement of γ-tocopherol by α-tocopherol in lipid membranes, resulting in decreased blood plasma γ-tocopherol levels [162].

Two independent studies evaluated the effect of the concomitant administration of vitamin E (as an antioxidant) and clomiphene citrate (as an antiestrogen) in patients with idiopathic oligoasthenozoospermia. The first (uncontrolled) study reported that after daily treatment for six months with vitamin E (400 mg) and clomiphene citrate (25 mg), idiopathic oligoasthenozoospermic patients (*n* = 30) had a significant improvement in the mean sperm concentration and in the mean total sperm motility [173]. The second (controlled) study was conducted on 60 infertile men with idiopathic oligoasthenozoospermia treated with the same administration protocol (400 mg/day of vitamin E and 25 mg day of clomiphene citrate for 6 months). A significant increase in sperm count and progressive sperm motility were again recorded. Interestingly, the improvement of semen parameters was mirrored by a significant increase in the pregnancy rate recorded at the end of the study (13.3% in the control and 36.7% in the group receiving drug treatment), corroborating the conclusion that the combination of an antioxidant and antiestrogen-based therapy is a valid option for the treatment of a selected group of men with unexplained isolated oligoasthenozoospermia [174].

As previously underlined, most of the studies focused their attention on the effectiveness of different vitamin E-containing antioxidant mixtures [138,139,140,141,142,143,144,158], thus rendering it difficult (if not impossible) to separate the effects of tocopherols from those of the other administered antioxidants. In this context, in a prospective, open-labeled, nonrandomized study conducted by Lipovac et al. [175], 299 subfertile males with at least one recent pathological semen analysis were treated for 3 months either with 500 mg L-carnitine/twice a day (*n* = 156) or with the combination of 440 mg L-carnitine + 250 mg L-arginine + 40 mg zinc +120 mg vitamin E + 80 mg GSH + 60 μg selenium + 15 mg coenzyme Q_10_ + 800 μg folic acid/once a day (*n* = 143). The authors reported that both groups, after the administration period, experienced a significant improvement in all studied sperm parameters (volume, density, overall progressive motility); however, relative changes of sperm density and overall progressive motility (including fast motility) were found to be higher in the group receiving the combined micronutrient treatment. In a different study, Gvozdjáková et al. [176] enrolled 40 infertile men with oligoasthenozoospermia who were treated with a combination of 440 mg L-carnitine fumarate + 30 mg ubiquinol + 75 IU vitamin E + 12 mg vitamin C twice a day for the first 3 months and once a day for the remaining 3 months of treatment. The authors reported a significant increase in the concentrations of total coenzyme Q_10_ (ubiquinone + ubiquinol) and α-tocopherol in blood and seminal plasma of treated patients. Furthermore, a significant increase in sperm density, a decrease of the sperm anomalies and a high pregnancy rate (45%) were measured after treatment [176]. Encouraging results were also obtained by Moslemi et al. [177] in a study performed on 690 infertile males affected by idiopathic asthenoteratozoospermia, who received daily oral supplementation of 400 IU of synthetic α-tocopherol (as a protector of cellular membranes from oxidative/nitrosative stress) in combination with 200 µg of L-selenomethionine (with the aim to increase the levels of the ROS scavenging enzyme Se-dependent GPx) for 14 weeks. After treatment, significant amelioration of sperm analysis parameters (sperm motility and morphology) occurred in 52.6% (362 cases) of patients, while 253 of them (36.6%) were unresponsive to the therapy. However, the authors reported that in 10.8% of patients (75 cases), spontaneous pregnancy was recorded. On the basis of these results, it was concluded that the combined administration of vitamin E and Se in cases of asthenoteratozoospermia is a potentially useful therapy not only to improve the quality of semen parameters but, most importantly, also to increase the probabilities to achieve normal pregnancy [177]. A summary of the clinical studies concerning the effects of α-tocopherol on male infertility is provided in Table 3.

### 3.4. Coenzyme Q_10_

Coenzyme Q (ubiquinone) is a fat-soluble compound ubiquitously found as a component of the mitochondrial electron transport chain (ETC) in oxygen-dependent living organisms. It is characterized by a redox-active benzoquinone head group that is conjugated to a poly-isoprenoid side-chain of species-specific length. Indeed, depending on the organism considered, the hydrophobic chain made up of isoprene units can be repeated from a minimum of 3 to a maximum of 10 units (as it occurs in humans). This last form of coenzyme Q, named coenzyme Q_10_ (CoQ_10_), is embedded in the mitochondrial inner membrane, actively participating in the ETC. Fully oxidized CoQ_10_ (CoQ_10ox_) accepts one couple of electrons from complex I and complex II and passing them to complex III, where the so-called Q-cycle takes place. In this cycle, during which four protons are pumped into the intermembrane space, reduced ubiquinol (CoQ_10_H_2_) is oxidized to ubiquinone (CoQ_10ox_), losing its electrons via two separate one-electron reductions. One of these electrons will serve to reduce the oxidized form of cytochrome c (anchored to a specific site of complex III protruding in the intermembrane space). The other electron left on the partially reduced radical semiquinone form of ubiquinone (CoQ^●−^) is internally transferred to cytochromes b_L_ and b_H_ and then used to reduce one molecule of CoQ_10ox_ (bound to the so-called Q_0_ site of complex III) to CoQ^●−^ [178]. The Q-cycle, during which from two CoQ_10_H_2_ molecules, one molecule of CoQ_10ox_ and one of CoQ_10_H_2,_ are finally obtained, represents a critical process during the ETC since electrons may leak and react with oxygen, forming superoxide and rendering the Q-cycle as one of the major sites of mitochondrial ROS production. Due to its ability to participate in one-electron transfer reactions, relative long-life, the low reactivity of its semiquinone radical, and its high hydrophobicity (clustering CoQ_10_ within biological membranes), an additional role of CoQ_10_H_2_ is to act as a potential free radical scavenger antioxidant. Indeed, it has been demonstrated that CoQ_10_ is involved in the protection against lipid peroxidation of low-density lipoproteins (LDL) (where it is oxidized before vitamin E), of mitochondria (where it counteracts the inhibitory effects of ETC substrates) and of plasma membranes (where it acts as an intermediate electron carrier) [179]. CoQ_10_ is considered as an endogenous antioxidant compound since its biosynthesis occurs in almost all cell types, and its abundance is not dependent on dietary supply. Due to its main localization, the final steps of CoQ_10_ biosynthesis are undertaken within the inner mitochondrial membrane and involve many regulatory components such as mitofusin 1 and 2, two proteins implicated in the mitochondrial fusion process [179,180]. The chemical features and the main biological role as a mobile electron transporter in the mitochondrial ETC render CoQ_10_ strictly clustered within mitochondria and poorly abundant in human body fluids, with concentrations ranging between 0.152 to 0.939 µmol/L in human serum [113,181] and between 0.0019 to 0.013 µmol/L in human seminal plasma [113,182]. However, since spermatozoa are particularly rich in mitochondria and require a relevant amount of ATP in order to correctly satisfy their energy demand, CoQ_10_ seems to play an important role in the physiology of these germinal cells. Furthermore, the well-established implications of oxidative/nitrosative stress in the insurgence of male infertility render CoQ_10_ potentially involved in the pathophysiology of this condition.

In patients affected by varicocele, oligozoospermia, asthenozoospermia or idiopathic infertility, decreased levels of CoQ_10_ in seminal plasma were measured when compared to control fertile subjects [182,183,184]. Notwithstanding the complexity of the process of absorption of CoQ_10_, caused by its chemical features and by problems linked to the efficacy of the ubiquinol—ubiquinone formulations [185], the supplementation with exogenous CoQ_10_, both as a single compound and in combination with other substances, has widely been applied in the treatment of male infertility. The most effective results were obtained when administering CoQ_10_H_2_, not only for the improvement of morpho-functional semen parameters but also for decreasing oxidative/nitrosative stress. In this scenario, Safarinejad et al. [186] conducted a randomized, double-blind, placebo-controlled trial on 228 men with idiopathic oligoasthenoteratozoospermia, in which 50% of the patients were daily treated for 26 weeks with 200 mg of CoQ_10_H_2_ and the remaining 50% with placebo. The authors reported a significant improvement in sperm density, sperm motility and sperm strict morphology in the group treated with CoQ_10_H_2_ compared to the placebo group. Interestingly, semen parameters gradually returned to baseline values at 12 weeks after the drug-off period, even though still significant differences in sperm density and motility were recorded [186]. The administration of CoQ_10_H_2_ has further been applied in three different studies for the treatment of idiopathic oligoasthenozoospermia. In the first prospective randomized clinical trial, the effect of the administration of 200 or 400 mg/die of oral CoQ_10_H_2_ on semen parameters of 65 infertile men with idiopathic oligoasthenoteratozoospermia was tested. After three months of treatment, results showed significant amelioration of the sperm analysis (sperm count and motility) and of semen antioxidant status, with higher improvements found in response to a dose of 400 mg/day [187]. In the second case–control study, the same number of patients affected by idiopathic oligoasthenozoospermia (*n* = 65) were treated for three months with 200 mg/day of an unspecified form of CoQ_10_. The authors reported a significant improvement of sperm analysis parameters (sperm concentration, progressive motility and total motility) compared to the baseline levels recorded before the beginning of treatment. Furthermore, CoQ_10_ supplementation increased the total antioxidant capacity and the glutathione peroxidase levels, with a consequent reduction in ROS levels and in the sperm DNA fragmentation index [188]. Similar results concerning the amelioration in sperm concentration, motility, and antioxidant status, were also obtained in the third study, in which 35 infertile males with idiopathic oligoasthenozoospermia were administered 200 mg/day of CoQ_10_H_2_ [189]. In two independent studies, the effectiveness of the 200 mg/die dosage of CoQ_10_H_2_ was evaluated by performing the administration of the drug in two daily doses of 100 mg each. In the first retrospective study, Cakiroglu et al. [190] reported significant changes in sperm morphology and motility in 62 patients with idiopathic asthenoteratozoospermia who received 100 mg of CoQ_10_H_2_ twice a day for six months. In the second randomized, placebo-controlled study, carried out on 47 infertile males affected by idiopathic oligoasthenoteratozoospermia, Nadjarzadeh et al. investigated the effects of the abovementioned CoQ_10_H_2_ administration protocol for 3 months on the expression of catalase and SOD and on the F2-isoprostanes levels in seminal plasma [191]. Besides showing a significant positive correlation between CoQ_10_ levels and the percent of morphologically normal spermatozoa, results indicated increased activity of the aforementioned antioxidant enzymes, accompanied by a significant decrease of F2-isoprostanes levels in seminal plasma. The authors suggested that the supplementation of CoQ_10_H_2_ to infertile males with oligoasthenoteratozoospermia can attenuate oxidative stress in seminal plasma by increasing antioxidant enzyme activity, ultimately improving semen parameters [191]. Surprisingly, a significant improvement in sperm forward motility, sperm density and in total antioxidant capacity was also obtained by Festa et al. [192], who administered a lower dose of 50 mg CoQ_10_H_2_ twice a day, for 12 consecutive weeks, to 38 infertile males affected by varicocele. However, it is worth underlining that no pregnancies were recorded in this cohort of patients [192]. Comparing the studies cited above, the beneficial effects of CoQ_10_ supplementation on sperm parameters, in particular on sperm density and motility, seem to be independent of the dosage and the etiology of infertility. Safarinejad [193] conducted a study on 287 infertile men with idiopathic oligoasthenoteratozoospermia treated with 300 mg CoQ_10_ (unspecified form) twice a day for 12 months, with a follow-up of additional 12 months after CoQ_10_ discontinuation. The Author reported a dramatic improvement in sperm density, motility, and morphology for treatment periods longer than 6 months. At the end of the 12 months of CoQ_10_ supplementation, 70% to 100% increases in morpho-functional sperm parameters (density, motility, and morphology) were recorded. As a consequence of the improved semen quality induced by the administration protocol, high pregnancy rates (34.1%) were also observed when the CoQ_10_ administration time was protracted beyond the 6th month, suggesting that oligoasthenoteratozoospermia may effectively be treated by the prolonged use of CoQ_10_ [193].

In addition to the previously mentioned studies reporting the CoQ_10_ supplementation in combination with other compounds [138,141,142,143,175,176], Tirabassi et al. [194] conducted an observational study on 20 patients affected by idiopathic asthenozoospermia and daily administered with 200 mg CoQ_10_ and 2660 mg of D-aspartate for 3 months. Notwithstanding no increment of CoQ_10_ in the seminal fluid of treated patients was recorded, the authors reported a significant improvement of sperm kinetic parameters and concomitantly decreased levels of ^●^NO, peroxynitrites and nitrosative stress-related damages to DNA after 3 months of administration [194]. A summary of the clinical studies concerning the effects of CoQ_10_ on male infertility is provided in Table 4.

## 4. Conclusions

A summary of the data from the literature of the last ten years, concerning the effects of the administration of low-molecular-weight antioxidants (AA, NAC, α-tocopherol and CoQ_10_) to treat male infertility and discussed in the present review, is provided in Table 1, Table 2, Table 3 and Table 4. Surprisingly, only 30 clinical studies tested the effects of the administration of AA, NAC, α-tocopherol and CoQ_10_ (each of them alone or in combination with other antioxidants and/or vitamins and/or micronutrients) to infertile males. It is even more surprising that only 33.3% of these studies (10/30) included, as an outcome measure to determine the effects of the therapy, the pregnancy and/or live birth rates. Of these 10 studies, only 4 were case–control studies, and only 2 of them found improvement of the pregnancy rate in the group of antioxidant-treated patients compared to untreated controls. Additionally, of the 30 studies considered in this review, only 43.3% (13/30) were case–control studies, 66.7% (20/30) enrolled a number of patients higher than 40 and 40% (16/40) carried out the administration of a single antioxidant (3 AA, 2 NAC, 2 α-tocopherol and 9 CoQ_10_).

In addition to the aforementioned critical points, the difficulty emerging in determining the real effectiveness of antioxidant-based therapies to ameliorate male infertility may certainly be due to further factors, including (i) studies using the same antioxidant(s) enrolled infertile males affected by different pathological conditions, therefore rendering impossible the comparison of the results; (ii) when considering the same antioxidant, administration protocols were characterized, in the majority of cases, by different dosages and by different supplementation times. An additional, rather crucial limitation of the studies in which antioxidant-based therapies for the treatment of male infertility were tested is represented by the fact that patients enrolled in almost all of these studies were not selected on the basis of specific deficiency of one or more antioxidants in their seminal fluid. To our opinion, before starting any antioxidant administration, it should be highly recommended (if not mandatory) to determine the full antioxidant profile in the seminal fluid of infertile patients (using analytical methods allowing the quantification of the most important water- and fat-soluble antioxidants and avoiding applying the poorly informative measurements of the so-called “total antioxidant capacity”). Only after performing a precise quantification of the seminal fluid antioxidants of every single infertile male, a precise selection of the patients and of the antioxidant(s) to administer will be possible. With this approach, the probability of recording benefits in targeted antioxidant treatments aimed to replenish the seminal fluid with the deficient or decreased antioxidant(s) should sensibly increase. Furthermore, this personalized medicine approach should decrease any possible side effects that may arise when undiscriminating supplementations with casual antioxidant cocktails are performed.

The last observations are that: (i) almost no data have been published concerning the administration of carotenoids and/or flavonoids in the treatment of infertile males, notwithstanding several compounds of these two classes are currently utilized as side-therapies for the treatments of other pathological states characterized by increased oxidative/nitrosative stress; (ii) since mitochondria play a pivotal role in oxidative/nitrosative stress, it is presumable that, to obtain better results, antioxidant administration should be accompanied by selected drugs favoring mitochondrial functions and energy metabolism.

In conclusion, due to the aforementioned deficiencies showed when evaluating results of the studies using low-molecular-weight antioxidant administration to infertile males, it is not currently possible to establish, beyond any doubt, either whether the antioxidant-based therapies are effective in treating male infertility or what is the best candidate antioxidant to obtain positive results, or what is the group of infertile males more susceptible to have beneficial effects from this therapeutic approach. Further case–control and longitudinal studies, enrolling a larger number of patients, using similar administration protocols (including the type of antioxidant, dosage, mode and time of administration) and enrolling patients with the same clinical diagnosis of infertility, are needed to clearly define the usefulness of antioxidant administration to treat male infertility.

## Figures and Tables

**Table 1:** Summary of the main results obtained in the 10 papers focusing on the effect of AA administration for the treatment of male infertility.

Authors	Patients (Number and Type of Infertility)	Controls (Number)	Antioxidant(s) (Type(s), Doses and Administration Protocol)	Duration of Treatment (Months)	Improvement (Sperm Parameters, Pregnancy and Live Birth Rates)	No Improvement (Sperm Parameters, Pregnancy and Live Birth Rates)
Cyrus A. et al. [135]	46 = varicocele	69	AA 500 mg/day	3	Sperm motility and morphology	Sperm count
Rafiee B. et al. [136]	200 = increasing Body mass index	50	AA 1000 mg/day	6	Sperm concentration and motility	
Vani K. et al. [137]	120 = testicular dysfunction high rate of DNA fragmentation	No controls	AA 1000 mg/day for 5 day/week	3	Sperm motility, sperm total count and significantly decreased the number of morphologically abnormal spermatozoa	
Abad C. et al. [138]	20 = AT	No controls	AA (60 mg), L-carnitine (1500 mg), CoQ_10_ (20 mg), vitamin E (10 mg), vitamin B_9_ (200 µg), vitamin B_12_ (1 µg), zinc (10 mg) and selenium (50 µg)/day	3	Sperm concentration, motility, vitality and morphology parameters, DNA integrity and 5% pregnancy rate	
Gual-Frau J. et al. [139]	20 = grade I varicocele	No controls	AA (60 mg), L-carnitine (1500 mg), CoQ_10_ (20 mg), vitamin E (10 mg), vitamin B_9_ (200 µg), vitamin B_12_ (1 µg), zinc (10 mg) and selenium (50 µg)/day	3	Sperm count and DNA integrity	Concentration, progressive motility, vitality, and morphology
Magdi Y. et al. [140]	120 = severe OAT	No controls	AA (1 g), vitamin E (400 mg) and L-carnitine (2 g)/day	6	Sperm concentration, percentage of total motility and progressive motility	
Kobori Y. et al. [141]	169 = idiopathic OAT	No controls	AA (60 mg), CoQ_10_ (120 mg) and vitamin E (40 mg)/day	3 and 6	Sperm concentration and motility	Atypical sperm cell number and semen volume
Terai K. et al. [142]	15 = O and/or A	16 = administered with a Chinese herbal medicine	AA (1000 mg), L-carnitine (750.1 mg), zinc (30 mg), astaxanthin (16.05 mg), Co Q_10_ (90.26 mg), vitamin B_12_ (60.1 µg) and of vitamin E (150 mg) 3 times/day	3	Total motile sperm count	Semen volume, sperm concentration and sperm motility
Sadaghiani S. et al. [143]	50 = infertile O and A	No controls	AA (100 mg), CoQ_10_ (30 mg), zinc (8 mg), vitamin E (12 mg) and folic acid (400 µg)/day selenium (200 mg)/other day	3	Volume, morphology, motion, count, progressive motility and pH	
Steiner AZ. Et al. [144]	174 = O, A, T and DNA-fragmented patients	86	AA (500 mg), vitamin E (400 mg), selenium (0.20 mg), L-carnitine (1000 mg), zinc (20 mg), folic acid (1000 mg), lycopene (10 mg), and vitamin D (2000 IU)/day	3 and 6		Sperm morphology, motility, DNA integrity, no different pregnancy and live-birth rates

AA = ascorbic acid; asthenozoospermia = A; asthenoteratozoospermia = AT; oligoasthenoteratozoospermia = OAT; oligoasthenozoospermia = OA; oligozoospermic = O; teratozoospermia = T.

**Table 2:** Summary of the main results obtained in the 4 papers focusing on the effect of *N*-acetylcysteine (NAC) administration for the treatment of male infertility.

Authors	Patients (Number and Type of Infertility)	Controls (Number)	Antioxidant(s) (Type(s), Doses and Administration Protocol)	Duration of Treatment (Months)	Improvement (Sperm Parameters, Pregnancy and Live Birth Rates)	No Improvement (Sperm Parameters, Pregnancy and Live Birth Rates)
Jannatifar R. et al. [156]	50 = AT	No controls	NAC (600 mg)/day	3	Sperm count, motility and normal morphology, DNA integrity	
Barekat F. et al. [157]	15 = varicocele	20	NAC (200 mg)/day post-varicocelectomy	3	Sperm motility and 33.4% pregnancy rate	
Dattilo M. et al. [158]	84 = ART failures	No controls	NAC (250 mg), extract of opuntia fig fruits (100 mg), quercetin (0.05 mg), betalain (0.001 mg), vitamins B_2_ (1.4 mg), B_3_ (16 mg), B_6_ (1.4 mg), B_9_ (400 μg), B_12_ (2.5 μg), zinc (12.5 mg), and vitamin E (12 mg)/day	4	DNA integrity, spontaneous pregnancy rate (21%), pregnancy rate (47.6%), and live birth rate (39.3%) after a new ART attempt.	Total sperm count, fast motility and normal morphology rates
Elsedfy H. et al. [159]	20 = BTM (hypogonadotropic hypogonadism)	No controls	NAC (600 mg) and L-carnitine (2 g)/day	6		Increased sperm deformities

NAC = *N*-acetylcysteine; asthenozoospermia = A; asthenoteratozoospermia = AT; oligoasthenoteratozoospermia = OAT; oligoasthenozoospermia = OA; oligozoospermic = O; teratozoospermia = T.

**Table 3:** Summary of the main results obtained in the 7 papers focusing on the effect of α-tocopherol administration for the treatment of male infertility.

Authors	Patients (Number and Type of Infertility)	Controls (Number)	Antioxidant(s) (Type(s), Doses and Administration Protocol)	Duration of Treatment (Months)	Improvement (Sperm Parameters, Pregnancy and Live Birth Rates)	No Improvement (Sperm Parameters, Pregnancy and Live Birth Rates)
Zerbinati C. et al. [162]	10 = sperm analysis anomalies and varicocele	No controls	α-Tocopherol (800 IU)/day	3		All parameters (data not shown)
Ener K. et al. [172]	22 = varicocele	23	Vitamin E (2×300 mg)/day post- varicocelectomy	12		Total sperm count, motile spermatozoa and no different pregnancy rates
El Sheikh MG. et al. [173]	60 = idiopathic OA	30	Vitamin E (400 mg) and clomiphene citrate (25 mg)/day	6	Means of sperm concentration and total sperm motility	
Ghanem H. et al. [174]	60 = idiopathic OA	30	Vitamin E (400 mg) and clomiphene citrate (25 mg)/day	6	Sperm count, progressive sperm motility and 36.7% pregnancy rate in treated patients vs. 13.3% in controls	Total sperm motility, percentage of abnormal forms and semen volume
Lipovac M. et al. [175]	143 = at least one recent (1 year) pathological semen analysis result	156	Vitamin E (120 mg), L-carnitine (440 mg), L-arginine (250 mg), zinc (40 mg), GSH (80 mg), selenium (60 μg), CoQ_10_ (15 mg) and folic acid (800 μg)/day	3	Volume, density, overall progressive motility (including slow and fast motility) and% of sperm with normal morphology	
Gvozdjáková et al. [176]	40 = OA	No controls	Vitamin E (75 IU), L-carnitine fumarate (440 mg), ubiquinol (30 mg) and AA (12 mg) twice a day for 3 months + once a day for 3 months	6	Sperm density, decrease of the sperm anomalies and 45% pregnancy rate	
Moslemi et al. [177]	690 = idiopathic AT	No controls	Vitamin E (400 IU) and Se (200 μg)/day	3.5	Sperm motility, morphology (52.6% cases) and 10.8% pregnancy rate	Sperm motility and morphology (36.6% cases)

CoQ_10_ = oxidized coenzyme Q_10_; asthenozoospermia = A; asthenoteratozoospermia = AT; oligoasthenoteratozoospermia = OAT; oligoasthenozoospermia = OA; oligozoospermic = O; teratozoospermia = T.

**Table 4:** Summary of the main results obtained in the 9 papers focusing on the effect of CoQ_10_ administration for the treatment of male infertility.

Authors	Patients (Number and Type of Infertility)	Controls (Number)	Antioxidant(s) (Type(s), Doses and Administration Protocol)	Duration of Treatment (Months)	Improvement (Sperm Parameters, Pregnancy and Live Birth Rates)	No Improvement (Sperm Parameters, Pregnancy and Live Birth Rates)
Safarinejad et al. [186]	114 = idiopathic OAT	114	CoQ_10_H_2_ (200 mg)/day	6.5	Sperm density, sperm motility and sperm morphology	Semen volume
Alahmar AT. [187]	65 = idiopathic OAT	No controls	CoQ_10_H_2_ (200 mg)/day (35 patients) CoQ_10_H_2_ (400 mg)/day (30 patients)	3	Sperm concentration, progressive motility and total motility (greater improvement after 400 mg/day dose)	
Alahmar AT. at al. [188]	65 = idiopathic OAT	40	CoQ_10_ (unspecified form, 200 mg)/day	3	Sperm concentration, progressive motility, total motility and DNA integrity	
Alahmar AT. et al. [189]	35 = idiopathic OAT	35	CoQ_10_H_2_ (200 mg)/day	3	Sperm concentration and progressive motility	Sperm morphology
Cakiroglu B. et al. [190]	62 = idiopathic AT	No controls	CoQ_10_H_2_ (100 mg) twice/day	6	Sperm morphology and motility	Sperm concentration
Nadjarzadeh A. et al. [181]	47 = idiopathic OAT	30	CoQ_10_H_2_ (100 mg) twice/day	3	Sperm morphology	Sperm concentration and motility
Festa et al. [192]	38 = varicocele	No controls	CoQ_10_H_2_ (50 mg) twice/day	3	Sperm forward motility and sperm density	No pregnancies
Safarinejad MR. [193]	287 = idiopathic OAT	No controls	CoQ_10_ (unspecified form, 300 mg) twice/day	12	Sperm concentration, motility percentage, total sperm count, morphology and 34.1% pregnancy rate	
Tirabassi et al. [194]	20 = idiopathic A	No controls	CoQ_10_ (unspecified form, 200 mg) and D-aspartate (2660 mg)/day	3	Total sperm motility	Sperm count and morphology

CoQ_10_H_2_ = reduced coenzyme Q_10_; CoQ_10_ = oxidized coenzyme Q_10_; asthenozoospermia = A; asthenoteratozoospermia = AT; oligoasthenoteratozoospermia = OAT; oligoasthenozoospermia = OA; oligozoospermic = O; teratozoospermia = T.

## Abbreviations

4-HNE	4-Hydroxynonenal
8-OHdG	8-Hydroxy-2′-deoxyguanosine
AA	Ascorbic acid
cAMP	Cyclic AMP
AVED	Ataxia with isolated vitamin e deficiency
CoQ_10_	Coenzyme Q_10_ or ubiquinone
CoQ_10 ox_	Oxidized coenzyme Q_10_
CoQ_10_H_2_	Reduced coenzyme Q_10_
DFI	DNA fragmentation index
DHA	Dehydroascorbic acid
eNOS	Epithelial nitric oxide synthase
ETC	Electron transfer chain
G6PDH	Glucose-6-phosphate dehydrogenase
GLUT 1, 3 or 4	Glucose transporters
GPx	Glutathione peroxidases
GR	GSH reductase
GSH	Reduced glutathione
GSSG	Oxidized glutathione
GST	Glutathione-S-transferase
IVF	In vitro fertilization
IL	Interleukin
iNOS	Inducible nitric oxide synthase
LDL	Low-density lipoproteins
MDA	Malondialdehyde
NAC	*N*-acetylcysteine
NADP^+^	Nicotinamide adenine dinucleotide phosphate, oxidized form
NADPH	Nicotinamide adenine dinucleotide phosphate, reduced form
NOS	Nitric oxide synthases
NOX5	NADPH oxidase, isoform 5
OXPHOS	Oxidative phosphorylation
PKA	Protein kinase A
PKC	Protein kinase C
PPP	Pentose phosphate pathway
PUFAs	Polyunsaturated fatty acids
RDI	Recommended daily intake
RNS	Reactive nitrogen species
ROS	Reactive oxygen species
SDI	Nuclear decondensation index
SOD	Superoxide dismutase
SVCT1 or 2	Sodium-dependent vitamin c transporter 1 or 2
α-TTP	α-Tocopherol transfer protein
WHO	World Health Organization
